# Physiological basis of vascular autocalibration (VasA): Comparison to hypercapnia calibration methods

**DOI:** 10.1002/mrm.26494

**Published:** 2016-11-09

**Authors:** Samira M. Kazan, Laurentius Huber, Guillaume Flandin, Dimo Ivanov, Peter Bandettini, Nikolaus Weiskopf

**Affiliations:** ^1^ Wellcome Trust Centre for Neuroimaging, UCL Institute of Neurology London U.K.; ^2^ Section on Functional Imaging Methods, National Institute of Mental Health Bethesda Maryland USA; ^3^ Maastricht Brain Imaging Centre Maastricht University Maastricht The Netherlands; ^4^ Department of Neurophysics Max Planck Institute for Human Cognitive and Brain Sciences Leipzig Germany

**Keywords:** vascular reactivity, vascularization differences, VasA, BOLD fMRI, BOLD calibration, SPM toolbox, VasA toolbox, autorescaling

## Abstract

**Purpose:**

The statistical power of functional MRI (fMRI) group studies is significantly hampered by high intersubject spatial and magnitude variance. We recently presented a vascular autocalibration method (VasA) to account for vascularization differences between subjects and hence improve the sensitivity in group studies. Here, we validate the novel calibration method by means of direct comparisons of VasA with more established measures of baseline venous blood volume (and indirectly vascular reactivity), the M‐value.

**Methods:**

Seven healthy volunteers participated in two 7 T (T) fMRI experiments to compare M‐values with VasA estimates: (i) a hypercapnia experiment to estimate voxelwise M‐value maps, and (ii) an fMRI experiment using visual stimulation to estimate voxelwise VasA maps.

**Results:**

We show that VasA and M‐value calibration maps show the same spatial profile, providing strong evidence that VasA is driven by local variations in vascular reactivity as reflected in the M‐value.

**Conclusion:**

The agreement of vascular reactivity maps obtained with VasA when compared with M‐value maps confirms empirically the hypothesis that the VasA method is an adequate tool to account for variations in fMRI response amplitudes caused by vascular reactivity differences in healthy volunteers. VasA can therefore directly account for them and increase the statistical power of group studies. The VasA toolbox is available as a statistical parametric mapping (SPM) toolbox, facilitating its general application. Magn Reson Med 78:1168–1173, 2017. © 2016 The Authors Magnetic Resonance in Medicine published by Wiley Periodicals, Inc. on behalf of International Society for Magnetic Resonance in Medicine.

## INTRODUCTION

The statistical power of functional magnetic resonance imaging (fMRI) group studies is significantly hampered by high intersubject variance, arising from differences in baseline physiology (ie, cerebral blood volume (CBV) and baseline deoxyhemoglobin concentration). In a pilot study, we recently presented a vascular autocalibration method (VasA) 1 to account for spatial vascularization differences between subjects, thus improving the sensitivity in group studies. VasA is based on the observation that global slow respiration‐induced blood oxygen level dependent (BOLD) signal changes within an fMRI experiment can be taken as an indicator for cerebral vascular reactivity (CVR) and baseline venous CBV. VasA calibration values can be obtained from any fMRI time series by estimating the power in low frequencies of the residuals in the task general linear model (GLM). These residuals can largely be assumed to be free from any task‐based variations and dominated by variations in breathing patterns.

In this study we further investigate the physiological basis of the VasA calibration maps, to better understand the potential limits of its application and its general robustness. First, we investigate the mechanism and the physiological basis underlying VasA by comparing it to the frequently used the Davis model calibration parameter M‐value as a gold standard 2. The M‐value is a function of the baseline‐CBV and venous‐deoxyhemoglobin concentration of the blood 3. Second, differences in cerebral vascular reactivity cause not only unwanted variability in studies across multiple participants, but can also induce unwanted variability across different brain areas of interest with different underlying vasculature 4, 5, 6. Here, we investigate the applicability of VasA calibration within individual brains across areas with different vascular reactivity (eg, areas enclosing large vessels compared with voxels with parenchyma only). We demonstrate that VasA reduces the undesired spatial variability.

## METHODS

### Study Design

Seven healthy participants (age range: 24–32 years, 3 females) were scanned for this study and approved by the local ethics committee. Written informed consent was obtained from all participants.

The first experiment consisted of a hypercapnia task of breathing air followed by 5% CO_2_ and then air (for 2 min, 5 min, then 5 min each), to estimate the M‐value. A premixed gas delivery with non‐rebreathing mask at a flow rate of 15 L/min was used. Heart rate and respiratory gas composition were monitored by a medical doctor in the magnet room and recorded with a BIOPAC MP 150 recording system (BIOPAC Systems, Goleta, California, USA). The system was calibrated before each experiment by means of three premixed gas mixtures with known relative volume fractions of O_2_ (20–95%), CO_2_ (0–5%), and N_2_ (0–80%).

**Figure 1:**
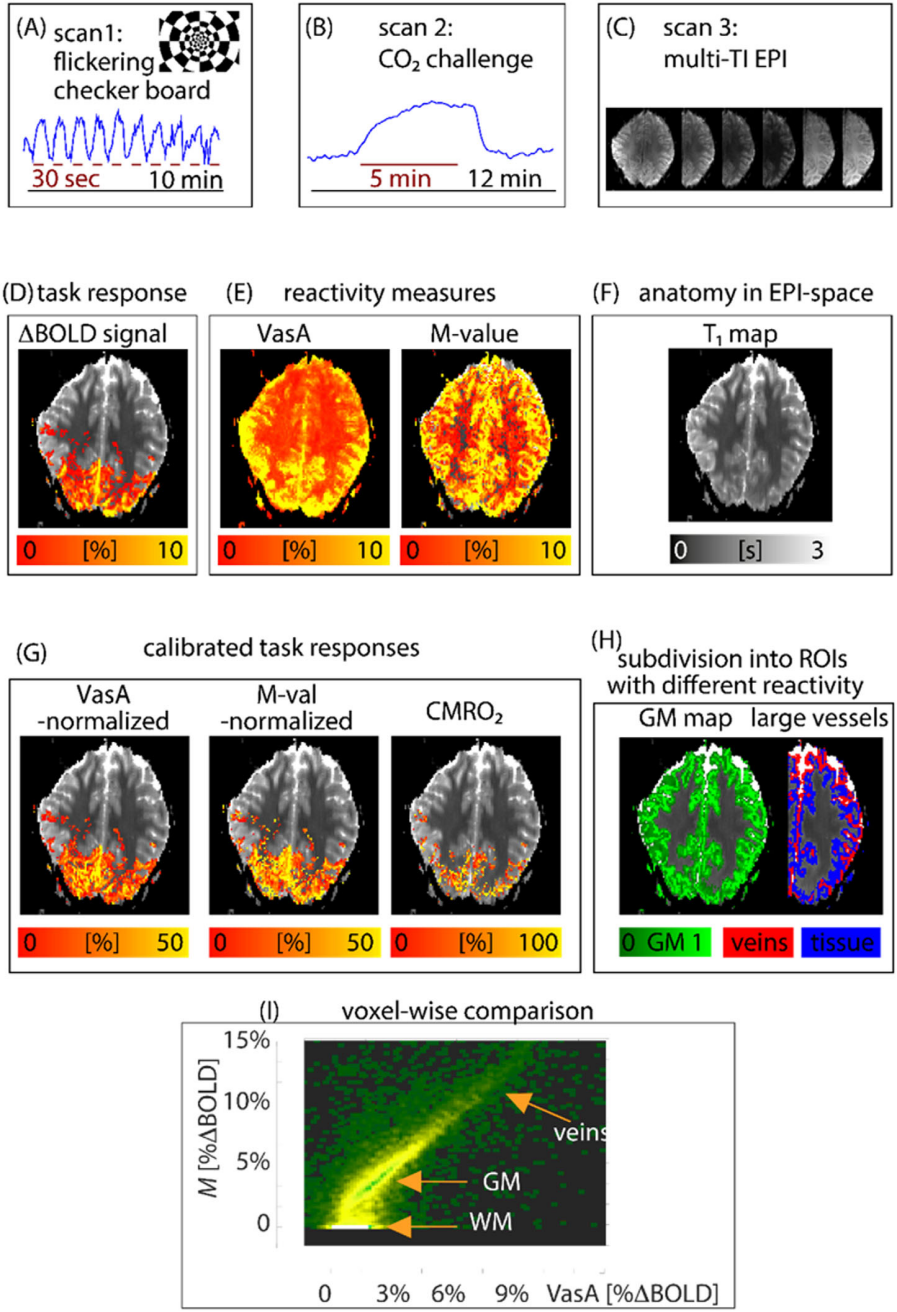
Evaluation procedure of the study (a–c) illustrated with a representative data set and example maps (d–f) along the analysis pipeline. The columns show which contrasts were obtained from which experiments. The right column in (c, f, h) demonstrates the data quality of the T_1_ maps in EPI space. (g) Calibrated task responses: normalized VasA, normalized M‐value, and CMRO_2_ maps. (The CMRO_2_ is shown for the sake of comparison.) (i) Scatter‐density plot of VasA with calibration M‐value parameter maps.

In the second experiment, one 10‐min flashing checkerboard paradigm for every participant consisting of 10 times 30‐s rest versus 30‐s stimulation was used to activate the visual cortex. The checkerboard pattern had a higher spatial frequency going toward the center of the visual field as shown in Figure 1a. The flickering frequency was 8 Hz (8 contrast reversals per second). The visual stimulus was projected from a projector into the bore of the MR scanner bymeans of a mirror system. The stimulus covered horizontally ≈ 27 º and vertically ≈ 21 º the visual field. During both experiments, vascular‐space‐occupancy (VASO) time series 7 of CBV changes and BOLD signal changes were captured with the SS‐SI‐VASO sequence 8.

### Data Acquisition

Data were acquired on a 7T Siemens MRI scanner (Munich, Germany) with the following protocol: 7 slices covering the visual cortex, echo time (TE)/T_I_/T_2_/repetition time (TR) = 19/765/2265/3000 ms, and adjusted B_1_‐independent inversion efficiency = 75% with custom‐designed TR frequency offset corrected inversion (FOCI) pulse variant, to avoid inflow of fresh blood in VASO 9. To minimize and assess the influence of partial‐voluming of gray matter (GM) with white matter (WM) and cerebrospinal fluid (CSF), and to be able to identify voxels with particularly large draining veins, a nominal isotropic resolution of 1.5 mm was used.

In addition to the functional scans, further inversion recovery measurements with multiple inversion times (TIs) of 36/200/300/900/1100 ms were performed with acquisition parameters otherwise identical to the functional scans for 21 s each. Following the methods of 10, these data were used to generate T_1_ estimates and GM maps with distortions identical to the functional data.

The M‐value was estimated with the Davis model 2 on a voxelwise basis. Calibrated BOLD studies are usually conducted with combined BOLD‐ASL sequences 11, and CBV values are calculated from cerebral blood flow (CBF) results based on the Grubb equation 12. However, at the desired high resolution of 1.5 mm, isotropic arterial spin labeling (ASL) CBF results are affected by the low contrast‐to‐noise ratio (CNR) of the ASL acquisitions 13, especially when no separate labeling coil is available. Hence, we followed the approach of high‐resolution CMRO_2_ mapping given in 14, and substituted CBF with CBV values in the Davis model using the inverse Grubb equation. As shown in 14, using VASO instead of ASL requires one minor adaption of the model: BOLD‐relevant CBV changes refer to deoxygenated “venous” compartments only, whereas VASO represents CBV changes along the entire vascular tree (containing “arterial” and “venous” compartments). The bias toward venous CBV in BOLD compared with global CBV estimates was described in 15 and can be addressed by the application of an additional venous Grubb coefficient 15. Following these studies and the parameters given in recent review articles 3, 16, 17, we assumed that α_total_ = 0.38 and α_veins_ = 0.2.

The parameter β in the Davis model can be considered as a model parameter relating $T_{2}^{*}$ to deoxyhemoglobin concentration. Because this value is field‐strength dependent, it should be adapted going from 3 T to 7 T. Considering that BOLD signal at 7 T is highly dominated by extravascular signal changes 18, we chose a value of β = 1.0 in agreement with previous 7T‐calibrated BOLD studies 13, 19, 20.

To relate quantitative VASO signal changes, which are inherently in units of mL CBV change per mL of tissue, into relative CBV changes, the baseline blood volume content must be assumed. Here, we chose CBV_rest_ = 5.5% following previous VASO studies 7, 8, 9.

### Data Processing and Map Estimation

Each subject's fMRI data were analyzed using Statistical Parametric Mapping (SPM 8; Wellcome Trust Centre for Neuroimaging, UCL, London) 21, implemented in MATLAB R2012b (MathWorks, Natick, Massachusetts, USA). Statistical analyses of the functional images were performed in two steps. In a first step, the fMRI data were analyzed using a standard mass univariate approach. First, a GLM was fitted to each voxel with a design matrix formed by convolving each subject's preprocessed fMRI time‐series function with a canonical hemodynamic response function (HRF) 21. The data were high‐pass filtered with a cut‐off period of 128 s and corrected for serial autocorrelations using a global autoregressive model of order 1.

In a second step, the difference between the spatially unsmoothed fMRI time series data and the GLM prediction were estimated from the residuals (VasA low‐frequency fluctuation maps), as detailed in 1. The residual time series at each voxel of the residuals map was Fourier transformed and the power spectrum was obtained. The averaged square root of the power within the frequency band of 0.01–0.08 Hz was then calculated at each voxel, as it best reflects the spontaneous respiration‐related CO_2_ and BOLD signal changes (see 1 for a detailed discussion). The resulting voxelwise map of low‐frequency fluctuation maps (ie, the VasA maps) was smoothed with an isotropic Gaussian kernel with 3‐mm full‐width‐half‐maximum (FWHM).

Regions of interest (ROIs) of stimulation‐induced activity were defined with the fMRI data as a cluster of voxels having *z*‐values above 2.3 and a significance level of *P* < 0.05 (corrected for multiple comparisons).

The estimated VasA and M‐value maps were coregistered for each participant.

Echo‐planar imaging (EPI) based T_1_ maps were used to classify all GM voxels into two categories: Voxels that encompassed the upper cortical layers and large draining veins versus voxels that did not contain larger draining pial veins following an approach similar to 8 (Figs. 1c, 1f, 1h). The separation algorithm relied on the assumption that at the 1.5‐mm resolution used, simultaneous partial voluming of any voxel with both WM and CSF did not occur. Because voxels with more CSF partial voluming would also have a higher probability of containing superficial cortical layers and pial vessels, these voxels were hereafter referred to as venous voxels, in contrast to deeper tissue voxels. The threshold of CSF partial voluming to separate the two subsets within the functional ROIs was adjusted such that the numbers of surface and deeper GM voxels were equal. This was done to avoid biases across subjects with different curvature.

## RESULTS

Figure 1 shows the overall evaluation procedure of the study using a representative subject and example maps along the analysis pipeline. The columns show which contrasts were obtained from which experiments. Note that in VasA normalization, both the CVR measure and the task response are obtained from the same data set. This is in contrast to conventional normalization methods that involve breathing manipulations separate from the task‐based fMRI experiment. The VasA CVR map appears to have a higher signal‐to‐noise ratio (SNR) compared with the M‐value map (Fig. 1e). A voxel scatter plot showing the relation of the two maps is given in Figure 1I. The right column exemplifies the data quality of the T_1_ maps in EPI space (Figs. 1c, 1f, 1h) and shows the corresponding ROIs of GM, and the ROIs with a different likelihood of containing large veins (red and blue ROIs in Fig. 1h), which defined the voxels used for the comparison.

**Figure 2:**
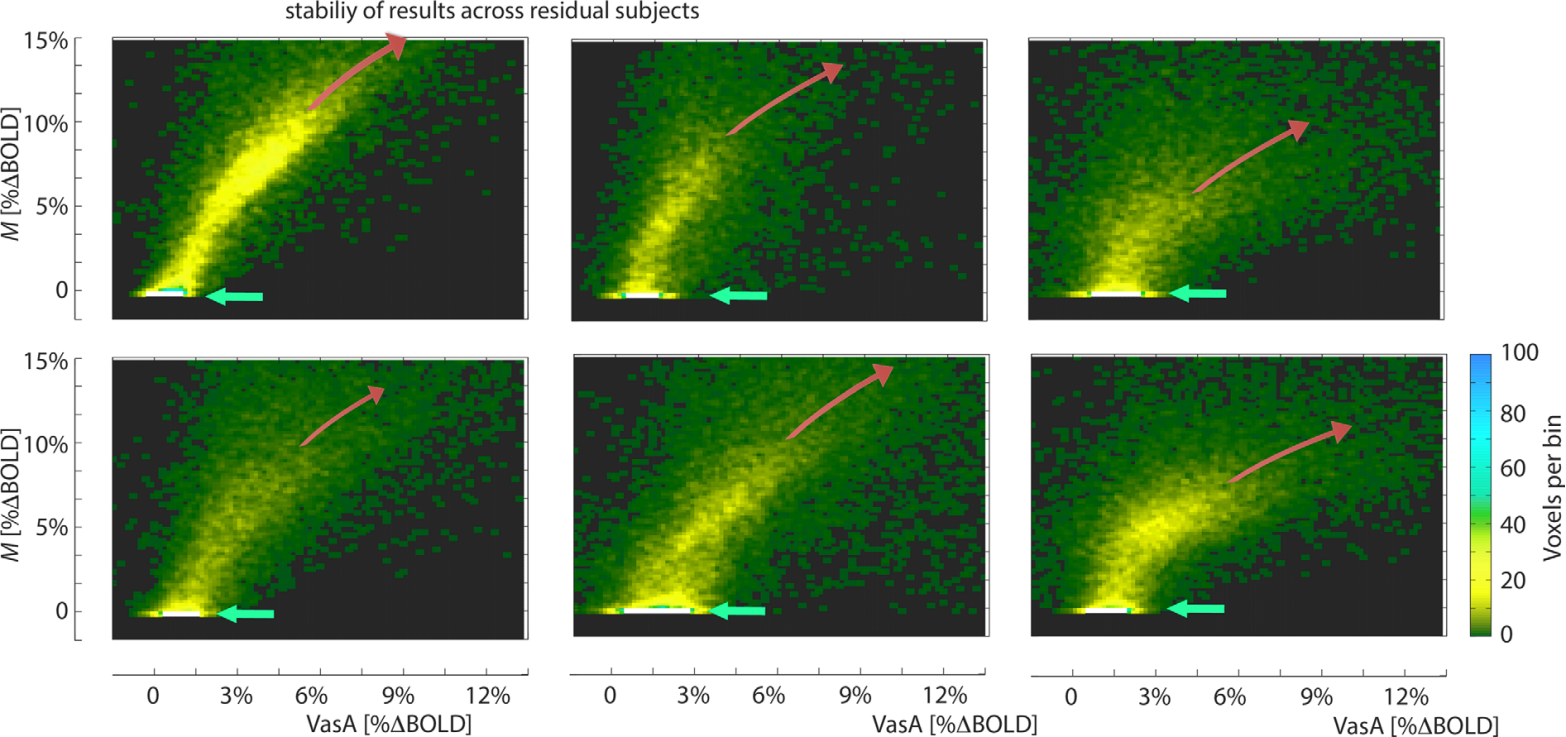
Scatter‐density plots of VasA maps and calibration M‐value parameter maps for four participants. VasA and M‐values are highly correlated. The curved red arrows indicate VasA overestimation in voxels with large physiological noise in CSF. White arrows indicate M‐value underestimation in white matter ROIs.

Figure 2 shows the scatter plots comparing VasA and M‐values across voxels as in Figure 1I for the remaining six subjects. It can be seen that VasA captured the range of vascular reactivity across voxels similar to the M‐value. The correlation coefficient (mean ± standard deviation) across all subjects was 0.44 ± 0.07. Correlation coefficients were Fisher *z*‐transformed for calculation of the mean and the standard deviation (and inverse transformed), to account for the non‐Gaussian distribution of correlation coefficients.

The vascular reactivity shown in the VasA map was relatively homogeneously distributed across the GM and not confined to the visual cortex, where most of the task response occurred (Fig. 1d).

VasA captured vascular reactivity well throughout large portions of GM, but there was a small tendency in VasA to overestimate vascular reactivity in regions of significant CSF partial‐voluming (defined in EPI space, based on multiple T_1_ maps) (Fig. 1e). This resulted in a nonlinear trend for high M‐values in the scatter plots (curved arrows in Fig. 2), which primarily reflected voxels containing pial veins close to CSF.

**Figure 3:**
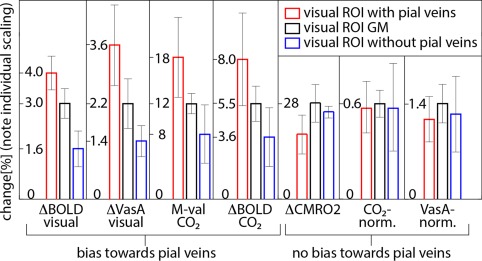
Results of various fMRI contrasts averaged across participants within the ROIs covering the visual cortex. Error bars indicate the standard deviation across participants. Different colors refer to different sub‐ROIs within the visual cortex. Red represents upper cortical layers at the border between GM and CSF with a high likelihood of containing pial veins. Blue represents deep cortical layers at the border of GM and WM with a reduced likelihood of containing pial veins. Black is linked to GM voxels without partial voluming with WM or CSF. fMRI signals without normalization are highest in ROIs containing pial veins (four leftmost diagrams). After VasA normalization, this bias is removed, similar to other more established normalization schemes (eg, CMRO_2_ normalization or CO_2_ normalization). Note the individual scaling of the *y*‐axis for each diagram.

Figure 3 illustrates the potential bias in measured BOLD response as a result of inhomogeneously distributed vascular reactivity, and the ability of VasA to account for it. Contrast maps of task‐induced BOLD signal change (based on a standard analysis and not VasA normalized), VasA normalization values, M‐values, and hypercapnia BOLD responses were significantly larger in ROIs that contained large pial veins. Applying VasA normalization homogenized the signal change to be more independent of large veins, similar to conventional normalization schemes.

**Figure 4:**
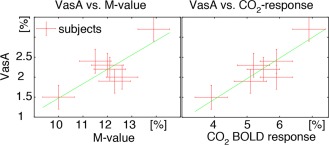
Scatter plots comparing VasA with other, more established measures of CVR; M‐value and BOLD response during hypercapnia, across participants. Subjects with the highest VasA value also show the highest values in the M‐value maps and hypercapnia BOLD response.

The correspondence between VasA and alternative measures of CVR across subjects is shown in Figure 4. Even though there is not much variability of CVR in the group of seven young healthy participants, VasA correlated well with the alternative CVR measures. Correlation coefficients were 0.80 for VasA versus M‐values, and 0.89 for VasA versus hypercapnia response, respectively.

## DISCUSSION

The strong correlation between VasA and M‐values suggests that they have similar physiological origins. The variations across participants are likely associated with variations in participants’ venous baseline oxygenation 22 and venous blood volume.

The M‐value maps in Figure 1e look noisier compared with the VasA maps. This might be associated with the fact that the M‐value estimation is much more prone to error propagation of less precise physiological measures like CBF and CBV, compared with BOLD 11. The feature that VasA appears less noisy compared with conventional M‐value maps is therefore likely the result of the higher precision of the BOLD effect measurements compared with the noisier CBV imaging method used to estimate the M‐value. M‐value normalization also relies on an additional step of coregistering results from two separate experiments, which might introduce an additional source of uncertainty compared with VasA normalization.

Across‐voxel scatter plots show some small differences between VasA and M‐values (Fig. 1I and Fig. 2). The deviations in areas of large partial voluming with WM could be the result of the difficulty in estimating M‐values with low‐SNR CBV data in WM. The deviations in areas of large partial‐voluming with CSF might arise from VasA overestimations, because of increased low‐frequency respiratory and cardiac noise contributions in CSF and its flow. VasA maps generally do not show increased bias in areas of large task‐related activation. This confirms that VasA measures veridically, reflecting vascular reactivity rather than residuals, as a result of imperfect modeling of task demands or residual nonmodeled neuronal activity.

Comparisons across voxel clusters with and without large draining veins reveal that VasA calibration, similar to M‐value calibration, can estimate and account for local variations in CVR across brain regions (Fig. 4).

The high correlation of VasA and M‐values across participants confirms the applicability of VasA in group studies to account for participant‐specific variations in CVR, as originally argued in 1.

In the data analysis of this study, we predicted the occurrence of large draining veins in GM wherever there was a large partial volume of CSF in GM. This approach has been developed and discussed previously in 23. It is based on the anatomical feature that the GM ribbon is drained with downstream veins sitting mostly above the cortical surface between GM and CSF 24, 25. At the interface between WM and GM, however, large draining veins are relatively rare. Based on this bias to find veins closer to CSF compared with WM, voxels with partial voluming of CSF and GM are considered “venous driven,” compared with voxels with partial voluming of WM and GM.

This approach is only applicable when the nominal resolution is smaller than the cortical thickness. As soon as there is a partial voluming effect of more than three compartments (eg, WM, GM, CSF), the model breaks down and relative CSF and venous contributions would be underestimated. Because CSF and WM voxels are only 2–4 mm apart, care must be taken that the anatomical MRI data used for estimating the partial volume effects match the functional data well. To minimize corresponding errors of coregistration and distortions, we used a distortion‐matched inversion recovery EPI sequence with identical distortions and geometry as the anatomical reference 26.

Future validation studies going to higher resolutions toward single‐vessel fMRI 27, such as in animal models, could help to investigate the accuracy of these assumptions.

As discussed in 1, VasA primarily accounts for signal variance that is associated with CO_2_‐related vascular reactivity. It does not model appropriately other sources of signal variation or noise, such as tasks unrelated to neural activity, potentially confounding the VasA estimates. Although it is rather unlikely that these effects induce false positives or reduce the sensitivity below the conventional analysis approach as previously discussed and shown in healthy volunteers 1, systematic differences caused by pathology may exacerbate these issues. For example, resting state activity levels may be systematically different between patient and control groups and lead to biased VasA adjustments. In contrast, VasA may also help to reduce the bias resulting from different vascular reactivity in pathology. Thus, we advise caution when applying VasA in these cases.

## CONCLUSIONS

We found a strong correlation between vascular calibration measures obtained with VasA and the more established vascular reactivity value M. This suggests that VasA‐calibration maps reflect physiological variability, particularly the baseline venous CBV distribution, and appear not to be affected by residual task‐related BOLD responses or other potential contaminations. Thus, they provide a reliable estimate for normalization of BOLD responses in fMRI group studies. They also offer a higher SNR compared with the conventional M‐value calibration method. To facilitate the use of VasA, we developed an SPM toolbox that allows for easy integration of VasA in the fMRI analysis workflow.
